# Multidisciplinary approaches to understanding collective cell migration in developmental biology

**DOI:** 10.1098/rsob.160056

**Published:** 2016-06-08

**Authors:** Linus J. Schumacher, Paul M. Kulesa, Rebecca McLennan, Ruth E. Baker, Philip K. Maini

**Affiliations:** 1 Mathematics, University of Oxford, Oxford, UK; 2 Department of Life Sciences and Centre for Integrative Systems Biology and Bioinformatics, Imperial College, London, UK; 3 Stowers Institute for Medical Research, 1000 E 50th Street, Kansas City, MO 60114, USA

**Keywords:** collective cell migration, mathematical modelling, developmental biology, neural crest

## Abstract

Mathematical models are becoming increasingly integrated with experimental efforts in the study of biological systems. Collective cell migration in developmental biology is a particularly fruitful application area for the development of theoretical models to predict the behaviour of complex multicellular systems with many interacting parts. In this context, mathematical models provide a tool to assess the consistency of experimental observations with testable mechanistic hypotheses. In this review, we showcase examples from recent years of multidisciplinary investigations of neural crest cell migration. The neural crest model system has been used to study how collective migration of cell populations is shaped by cell–cell interactions, cell–environmental interactions and heterogeneity between cells. The wide range of emergent behaviours exhibited by neural crest cells in different embryonal locations and in different organisms helps us chart out the spectrum of collective cell migration. At the same time, this diversity in migratory characteristics highlights the need to reconcile or unify the array of currently hypothesized mechanisms through the next generation of experimental data and generalized theoretical descriptions.

## 1. Introduction

Developmental biology strives to understand how a complex organism builds itself from a single cell. Cell migration plays an important role in the development of multicellular organisms, as it facilitates targeted bulk movement of cells. This can take the form of a sheet of cells moving and deforming during, for example, gastrulation, or cells migrating over long distances to their eventual positions within the embryo as, for example, in neural crest cell migration. Thus, the study of collective cell migration promises to provide a key to understand the vastly different morphologies observed between closely related vertebrate species. In addition, there is a translational motivation to unravel the mechanisms of collective cell migration, for example to understand regulation in wound healing, when cells move to close a breach, and because severe consequences can arise when cell migration is mistargeted, resulting in developmental defects [3], or uncontrolled, as is the case in metastatic cancer [19].

The remarkable process of organismal development involves many interacting parts, both at the molecular and cellular level, and the identity and organization of these parts change over time as the embryo grows. This dynamic complexity, as well as the ever-increasing availability of quantitative data, make developmental biology fertile for interdisciplinary contributions. Collective cell migration in particular represents an opportunity for interdisciplinary approaches as it can exhibit emergent, non-intuitive outcomes. Verbal reasoning and linear thinking often cannot compute the outcome of many complex, generally nonlinear interactions. Mathematical models, which are quantitative and logically rigorous representations of the conceptual models already present in the researcher's mind, let us quickly probe many hypotheses for consistency with experimental observations. Hypotheses generated from a mathematical model often stem from a study of how the prevailing model fails, and in this sense, mathematical models are most useful not to show what can be, but to show what cannot be. When this hypothesis generation and testing process is integrated into an iterative predict–test–refine cycle, mathematical models and their computational implementations help to accelerate biological discovery, and are thus becoming another staple in the suite of tools available to researchers in biology, along side animal, *in vitro* and verbal models.

In this review, we first consider the minimal (theoretical) requirements for movement of cell populations and the characteristics of collective migration. Then, we showcase the interplay of theory and experiment through specific examples of collective cell migration, focusing on the neural crest as a cell population with a wide range of collective migratory characteristics. We compare and contrast current complementary hypotheses in the field, and discuss how generalized models may help us to understand these as realizations of an overarching theory.

## 2. Collective cell migration

To begin with the basics, let us consider the minimal theoretical requirements for the collective movement of cell populations. At an abstract level, these are a global displacement of the cell population and local interactions between cells to correlate their movement, and to mediate cohesion and dispersal. A third ingredient, interactions between cells and their environment, is also required for basic motility. These interactions may also influence the population behaviour, for example through directional signals, or the boundary (outer surface) of the environment within which the cells are moving may be impermeable, thus confining the cells to stay within the domain. Thus, collective motion of cells is characterized by a display of coordination of movement at the tissue scale, which emerges from local interactions between individual cells and their environment. Such self-organization is familiar from the collective behaviour of groups of animals [85], although the interactions of cells are restricted to a more limited variety of sensory modalities.

### 2.1. Neural crest as a model system for collective cell migration

**Figure 1.**
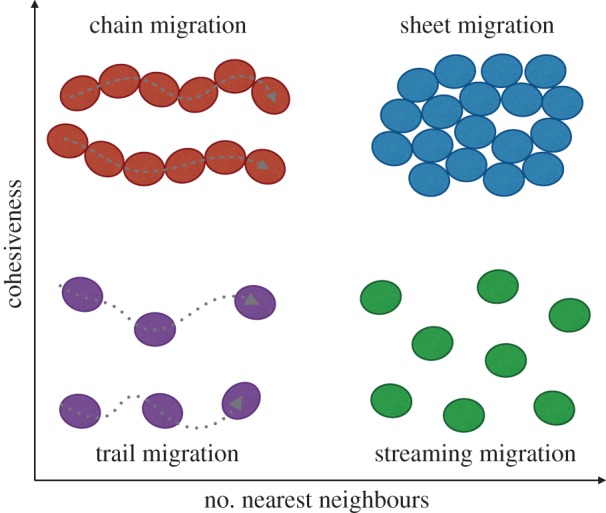
Conceptual drawing of the spectrum of collective cell migration. Different morphologies of collective cell migration can be characterized by their cohesiveness during migration (inversely related to density), as well as the number of nearest neighbours with which a cell interacts while moving (i.e. the topological arrangement of individual cells in the population). Cells (ellipses) can migrate in linear chains (top left), with persistent contact to cells either side of them, or along trails formed by preceding cells (bottom left). In migrating sheets, cells may maintain most of their nearest neighbours over time (top right), whereas in streaming migration cell–cell contacts occur at longer range and with potentially frequent neighbour rearrangement (bottom right). These concepts easily extend to three-dimensional migration, in which case the place of migrating sheets can be taken by moving clusters or spheroids.

A remarkable example of long-distance, coordinated, directed migration of eukaryotic cells is found in the neural crest. Neural crest cells are an important migratory population of cells in vertebrate embryonal development. They emerge and migrate away from the dorsal neural tube, a structure that develops into the brain and spinal cord, in a head-to-tail manner. Neural crest cells are sculpted into discrete streams that follow stereotypical pathways [35]. As multipotent cells, neural crest cells contribute to a variety of tissues in different parts of the body, such as the peripheral nervous system, structures in the head and heart, and many others [32,42]. The neural crest serves as a model system to study sheet, chain and streaming cell migration (figure 1), and is thus particularly useful for advancing our understanding of the spectrum of collective cell migration. Our own recent efforts have investigated the effect of population heterogeneity on collective migration [49,50], as well as the plasticity versus predetermination of cell states and migratory routes [51].

Diseases associated with defects in neural crest cell biology are known as neurocristopathies [3]. Neurocristopathic malformations include cleft lip, unusual pigmentation and abnormal ear development [80], as well as conditions such as Hirschsprung's disease, which is a lack of nerves in part of the gut [40,41]. Understanding the mechanisms of neural crest cell motility and guidance can aid in developing preventative and restorative treatments of neurocristopathies. The neural crest also provides a potential model system to study cancer metastasis. The neural crest lineage is the origin of the cancers melanoma and neuroblastoma, and their metastatic invasion may resemble the migratory characteristics of embryonic neural crest cells. When metastatic melanoma cells are transplanted into the neural crest microenvironment in the developing chick embryo, they do not form tumours, and some of the transplanted melanoma cells migrate along the host neural crest's path and into target tissues in the head and trunk [2,22,36]. Thus, understanding neural crest cell behaviour may not only shed light on the migratory characteristics of the metastatic phenotype of cancer cells, but also the mechanisms underlying its plasticity, as controlled by the embryonic microenvironment. Understanding these mechanisms holds the potential to develop strategies to revert the metastatic phenotype and reprogramme cancer cells [22,38].

Static cell labelling and dynamic *in vivo* imaging studies have shed light on neural crest migratory patterns across a wide variety of vertebrate embryo model systems. Early tracing studies that mapped cell positions over time in embryo models such as the chick [70,86], mouse [69,71], zebrafish [67], *Xenopus* [31] and axolotl [44] confirmed many spatio-temporal similarities of the neural crest migration pattern. In more recent years, *in vivo* time-lapse analyses have revealed an exciting variety in individual and group neural crest cell behaviours that are dependent on extracellular influences. These cell behaviours include migration in multicellular streams and chain-like arrays [34,89] with long filopodial protrusions during cell-to-cell contact [33,47,92], sheet migration [1], proliferation [53,64,74] and contact inhibition of locomotion (CiL) [5]. Advances in using lipophilic dyes in lamprey [58], snake [62] and turtle [7] have shown similarities to the overall neural crest migratory pattern, but some subtle differences exist in the timing of migration of different neural crest cell subpopulations. Thus, current and emerging data on neural crest migratory patterns in a large number of vertebrate embryo model systems can be exploited to better understand underlying cell migration and patterning mechanisms.

It is important to note that the characteristics of migration differ between neural crest cells at different axial levels in one embryo. For example, chick neural cells that emerge from the third to the fifth rhombomeres (r, mid-r3 to mid-r5) form into a stream adjacent to r4 in a loose arrangement, with frequent contact through filopodial extensions but non-constant neighbour relationships [78] (box 1). At the level of r1 and r7 [34], as well as in the trunk of the chick embryo [26], migration may proceed in linear chains. Whether there are universal mechanisms underlying this variety of behaviour is subject to ongoing research, and unified theories of neural crest migration across different model organisms remain the subject of future work.

Box 1. **Multicellular streaming.**

Some migratory groups of cells, for example chick cranial neural crest cells, are made up of individuals that autonomously control their motility, yet nevertheless rely on cell–cell contacts for group navigation. This type of migration has been termed *loose* (as opposed to cohesive) collective cell migration [66] as well as *multicellular streaming* [19]. Multicellular streaming has not been consistently classified as collective migration in the literature, which typically focuses on the movement of confluent sheets, persistent chains and cohesive spheroids. We, and others [66], argue that multicellular streaming can be considered *collective* in the wider sense of collective behaviour of individual agents, as studied in many other systems [85], and should therefore be included in the definition of collective cell migration.

## 3. Guidance mechanisms

**Figure 2.**
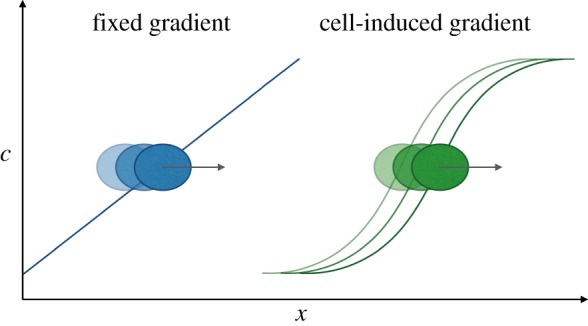
Schematic of chemotaxis of a cell up a fixed (left, blue) and cell-induced, or self-generated, gradient (right, green) of chemoattractant. Lines show the concentration (*c*) of chemoattractant along space (*x*), in which cells (ellipses) migrate. Darker shapes illustrate successive time-points.

Moving a group of cells from one place to another requires either global directional signals, to which each cell responds individually (though the overall response can differ from the sum of individual responses), or local signals that translate to a population response through interactions between cells. These signals come in a variety of modalities, such as chemical [73], mechanical [84] or electrical [46]. A simple and often studied guidance mechanism of cell migration is chemotaxis up or down a gradient of an attractive or repulsive cue (figure 2). In bacterial chemotaxis, for example, this cue might be provided by the naturally occurring distribution of food or toxins. In the development of complex eukaryotic organisms, gradients of chemicals known as morphogens are thought to direct growth, movement and differentiation [65]. Morphogen gradients are often thought of as pre-existing, requiring additional mechanisms to establish them prior to migration, and many such mechanisms are known or hypothesized [55]. An alternative concept to guidance through fixed gradients is the dynamic interpretation and generation of gradient signals during migration.

### 3.1. Cell-induced gradients with leader–follower heterogeneity

In the absence of a pre-established gradient of chemoattractant to guide migration (e.g. if a cue is uniformly distributed), an alternative guidance mechanism is provided by the cell-induced (or self-generated) gradient hypothesis (figure 2) [32,49,76]. In this model, cells bind and internalize the attractive cue. Through this local consumption of chemoattractant, a gradient is created that the cells can follow. If the induced gradient is locally symmetric around a cell, then breaking of the symmetry is required to initiate migration. The symmetry in the local chemoattractant gradient can be broken in a number of ways, for example by the initial velocity of the cells, or by cells entering the migratory domain from one side. The cell-induced gradient mechanism may cease to work in cases where chemoattractant diffusion is fast enough (or chemoattractant consumption low enough) to flatten out the chemoattractant profile before cells can sense and respond to a local gradient. Cell-induced gradient migration in developmental biology has been studied in the zebrafish lateral line primordium [76] and chick cranial neural crest [32,49,50]. In cancer, locally created chemotactic gradients have also been suggested to drive the dispersal of metastatic melanoma cells [54].

Another alternative to chemotaxis along pre-existing chemoattractant gradients is starvation-driven dispersal, in which cells move randomly but increase their speed when a relevant chemical resource is low. Models of starvation-driven dispersal [91] have been shown to give similar results to classic models of chemotaxis [27,28]. In eukaryotic cell populations, we are unaware of cases where the differences in cell speeds are as large as those required by the starvation-driven dispersal mechanism.

Box 2. **Heterogeneity versus parsimony.**

A physicist, mathematician or biologist may wonder: ‘Why do we need leader and follower cells? We know that collective behaviour can arise from identical agents, which is simpler.’ This is a valid concern. However, nature has not necessarily found the ‘simplest’ (most parsimonious) solution for every instance of collective cell migration. Evidence in the literature clearly shows clues to functional population heterogeneity. These observed differences between cell properties and behaviour could be an artefact of finitely sized systems, or a consequence of confinement. For example, proliferative ‘superstars’ in front-driven neural crest cell migration have been argued to necessarily arise through competitive growth in a confined environment [75]. This line of thought would lead us to consider that cells are identical, and the observed heterogeneity is really a result of dynamic responses to local differences in the environment. In our view, this distinction is largely semantic, and the above perspective very much compatible with the use of the terms ‘leaders’ and ‘followers’ as useful descriptors of cell migratory states. In combination with single cell genetic profiling, mathematical models can help to determine whether these descriptors are best thought of as discrete states or continuously varying. In addition, there is untapped potential to translate concepts and methods from the study of animal collective behaviour [12,56]. As an example, automated statistical analysis of cell-tracking data [72] can help us determine leading cells through temporal cross-correlation [56] or information theoretic measures [63]. Even between independently migrating cells, however, a certain degree of correlation is to be expected by chance, and therefore any statistical approach to detect leading cells needs to be compared with the appropriate null hypothesis, such as all cells behaving identically but with a certain amount of intrinsic noise.

Box 3. **Contact inhibition of locomotion and volume exclusion.**

**Figure 3.**
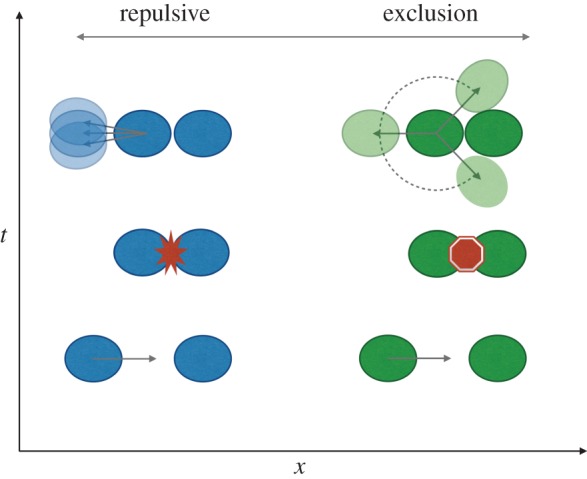
Schematic of the spectrum of cell–cell interactions, from repulsive interactions (left, blue) to volume exclusion (right, green). With repulsive interactions, cells move away from the point of contact with another cell (second cell shown as stationary for simplicity). With volume exclusion, cells block each other's movement, but can move to any space not occupied by another cell.

When considering the effects, such as CiL, that one cell has on another cell's movement, we can define a spectrum from repulsive to volume-excluding interactions (figure 3). Repulsive interactions imply adopting a direction of movement that is biased away from the point of contact with another cell, while exclusion allows movement into any nearby space that is unoccupied by other cells. In the case where the direction of movement after a cell–cell encounter is chosen uniformly from the unoccupied space, that is, as the bias away from the point of contact goes to zero, a CiL-like mechanism may become indistinguishable (at the population scale) from the purely physical phenomenon of volume exclusion (the fact thattwo cells cannot occupy the same space). The importance of volume exclusion in cell migration has been studied by deriving continuum descriptions of moving cell populations [16], including the effects of different types of volume exclusion [17], such as when in the movement a cell stops owing to the sensing of another cell. The difference in outcome between repulsive CiL and directionally unbiased volume exclusion has been studied in simulations of haemocyte dispersal [13], where volume exclusion fails to produce the periodic patterning that results from dispersal through repulsive CiL. Similar computational experiments have not been carried out in models of neural crest-cell-directed migration. This presents a promising avenue for future work to generalize complementary descriptions of collective cell migration in different model organisms (such as chick, *Xenopus* and zebrafish), and to investigate whether we can distinguish between CiL and volume exclusion from currently available *in vivo* data on neural crest cell migration.

In many systems exhibiting collective cell migration, a degree of functional population heterogeneity can be observed, a common example being the distinction between leader and follower states with ‘a clear division of labour’ [66]: leader cells read out directional information (e.g. from environmental signals), whereas follower cells instead obtain their directional cues from the leader cells, through secreted signals, mechanical sensing, pulling or tracks in the extracellular matrix (ECM) [68], for example. In principle, these differences between cells could be pre-specified, emerge from intercellular interactions, or be induced by signals in the tissue environment. Evidence for leader–follower heterogeneity can be found, for example, in angiogenesis (tip and stalk cells), zebrafish lateral line primordium [76], *Drosophila* border cells [24] and chick cranial neural crest cells [49]. Another recent example is that of neutrophils guiding T-cells using ‘breadcrumb’-like trails of chemokine [43]. For a review on this topic, see Khalil & Friedl [30], who discuss a range of leader cell morphologies and mechanisms of induction.

In the chick cranial neural crest system, vascular endothelial growth factor (VEGF) has been shown to act as a chemoattractant *in vitro* and *in vivo*, and is hypothesized to guide cells through a cell-induced gradient. VEGF is expressed in the ectoderm of branchial arch 2 (ba2), overlying the neural crest migratory route [52]. The expression of VEGF has been observed to be spatially uniform in the tissue up to the entrance of ba2, so there does not seem to be a pre-existing gradient prior to cranial neural crest cell migration [52]. There is emerging evidence that cell-induced migration is also employed by cardiac neural crest cells, where the role of VEGF is instead played by stromal cell-derived factor 1 (SDF1) (reviewed in [37]).

A mathematical model of chick cranial neural crest migration [49,50] was used to test the hypothesis arising from the aforementioned verbal model: VEGF is produced by the overlying ectoderm, cells emerge into the domain and, consuming the VEGF, create a gradient up which they move. This is a very seductive model, but when translated into a mathematical framework, it was shown that it could not recapitulate observed behaviours. Simulations showed that a single cell type responding to a gradient could not reliably migrate as a stream, as trailing cells can get stuck in a region of depleted chemoattractant. Importantly, this model also demonstrated that random movement (at the speeds measured *in vivo*) is insufficiently fast for invasion of the tissue on the relevant time scales, as cells would not migrate very far, even on a growing domain. The simulation framework was then used to test the hypothesis that cells at the front are leaders, responding chemotactically to VEGF, whereas the cells at the back are followers, responding to the leaders. This modified set-up allowed simulation of successful streaming migration, leading to the prediction of heterogeneity between the front and back of the neural crest population, which was validated by the experimental model. For this proof-of-principle result, the contingent of leaders made up the front 30% of the stream.

Further refinement of both the mathematical model and the gene expression profiling revealed that the group of cells in a leader state may be restricted to the ‘trailblazing’ cells at the most distal invasive front of the neural crest migratory stream [50]. Refining the mathematical model in line with current experimental evidence [49,50] made it possible to predict that streaming migration in a cell-induced gradient can be more efficient with fewer leader cells [50]. This guided single cell gene expression profiling experiments to identify a molecular signature of cells in the leader state, which are narrowly confined to the invasive front and which we thus termed ‘trailblazers’ [50]. When a single transcription factor upstream of several genes in this trailblazer signature was overexpressed in neural crest cells *in vivo*, the migration defect was just as predicted by the mathematical model when a larger number of leader cells was distributed throughout the stream (rather than only located an the invasive front): cells migrated just as far as in the unperturbed case, but at reduced population density. In addition, the mathematical and *in vivo* models were used to investigate how a leader cell state may be induced by the presence of VEGF (or a VEGF gradient), which revealed that the *in vitro* model was not representative of the *in vivo* heterogeneity of gene expression [51]. Finally, *in vivo* experimental knockdown^1^ [77] of VEGF signalling that was targeted to trailing cells showed no effect on their migration, supporting the hypothesis that trailing cells receive guidance information from leading cells or other signals [51].

### 3.2. Contact inhibition of locomotion and local attraction

A complementary mechanism to guidance through chemical gradients is the combination of CiL and co-attraction (CoA) [6]. In this mechanism, group cohesion is provided by the balance of repulsion and attraction between cells. CiL promotes dispersal, whereas CoA balances the dispersion that would otherwise result from CiL alone. This mechanism can be thought of as an effective potential resulting in repulsion at short range and attraction at intermediate ranges, leading overall to the cohesive (and potentially persistent) but undirected collective motion of a group of actively moving cells. Directionality of the overall migration can be provided by confinement or directional signals, such as chemoattractant gradients. Together, CiL and CoA have been suggested as a general mechanism for collective neural crest cell migration. Evidence for this has been found in *Xenopus* and zebrafish, where cranial neural crest cells acquire polarization through inhibition of membrane protrusions at intercellular contact sites, in combination with promotion of protrusions at free edges [79], which are thought to be stabilized and amplified through SDF1 [80]. In the *Xenopus* system, CoA is mediated by the peptide Ca3 and its receptor Ca3R, which are expressed by migrating neural crest cells [60].

The role of CiL with CoA has been explored in computational models integrated with experiments using the *Xenopus* neural crest system [6,87]. Using a force-based model of cell movement, a balance of attractive (CoA) and repulsive forces (CiL) was found to promote cohesive movement of a group of agents, whereas CoA alone led to aggregation, and CiL by itself resulted in dispersal. The simulated migration can be persistent, yet overall directionality has to be given (as in other systems) by a directional signal or confinement, for example through restrictive boundary conditions on the computational domain.

While CiL paired with CoA creates a mechanism for group cohesion and alignment of motion between cells, it still requires an overall directional signal to enable long-range navigation in the embryo, such as a chemoattractant gradient. It is therefore not mutually exclusive with the cell-induced gradient model [49]. In the context of the cranial neural crest in *Xenopus*, Theveneau *et al*. [81] propose a ‘chase-and-run’ hypothesis, in which neural crest cells are attracted to placode cells via SDF1, and placode cells are repulsed on contact through planar cell polarity (PCP) and N-cadherin signalling. While this mechanism has yet to be observed in other neural crest model systems, it suggests further studies that simultaneously visualize neural crest–placodal interactions. In contrast, placode assembly in chicks may be independent of interactions with the neural crest [81], and studies later in development suggest that neural crest cells then guide (rather than repel) neuron growth from placodes [18].

Whether CiL is relevant for the collective migration of cranial neural crest in other model systems remains to be confirmed. Chick neural crest cells have been observed to move in the same direction as each other following contact [78], suggesting contact guidance rather than contact inhibition, though this could conceivably be a function of cell density. Local attraction between cells through secreted factors may play a role in other neural crest systems, but the corresponding molecules have not yet been identified. Further differences in neural crest migratory mechanisms between different organisms are still being discovered. For example, PCP signalling, which is involved in CiL, is required for neural crest cell migration in *Xenopus* and zebrafish [5], but not in mice [61], at least for the particular class of PCP tested. While the prospect of universal guidance mechanisms for neural crest cells in all vertebrates is enticing, we must acknowledge and appreciate the differences between biological model systems.

### 3.3. Proliferation-driven colonization

Instead of directed migration, colonization of tissue can also be driven through proliferative expansion, in which cell fronts advance through increased division of cells at the free edge of the cell population, filling in the space adjacent to the population through division rather than directed motion. In concert with spatial confinement, frontal expansion can provide a direction and thus facilitate invasion. Evidence for proliferation-driven colonization is found, for example, in the mouse gut, where enteric neural crest cells (for a dedicated review of experiments and models of enteric neural crest migration, see [57]) at the migrating front have higher proliferation rates than the rest of the population [41,74], which is hypothesized to accelerate the otherwise undirected migration in the absence of long-range directional cues. Evidence from experiments and simulations for a subgroup of more proliferative cells, dubbed ‘superstars’ [9], has given rise to the hypothesis that heterogeneity between cells may enhance this mode of collective cell migration, conceptually akin to the leader cell state in chick cranial neural crest migration. However, it has been argued that one will necessarily discover over-represented lineages in scenarios of competitive growth under confinement [75], raising the possibility that population heterogeneity in this context is an artefact resulting from a selection process on a population of identical dividing cells. This selection bias towards few lineages, its possible dependence on the initial state of the system and the effects on patterning have been further explored in recent studies of colonization of skin by melanocytes in mice [53]. This study also demonstrated the ability of colonization through random movement and proliferation to give rise to chimeric patterns, such as stripes and spots, typically associated with directed migration. This highlights the potential of different guidance mechanisms to interact in the spreading of cell populations, and the need to carefully disentangle the contribution of different mechanisms to effects such as patterning. In chick cranial neural crest cells [39], there is evidence for increased proliferation in the front portion of the migrating stream, though further investigations showed that proliferation may not come into play until cells reach their target site, the branchial arches [64], and thus contribute little to the invasive capabilities of the cell population.

## 4. Discussion

In this review, we introduced minimal requirements for collective cell migration, including directional signals, cell–cell interactions and cell–environment interactions. Drawing on examples from our own work and the related literature, we illustrated how integration of mathematical modelling with experiments has been used to increase our understanding of the various mechanisms contributing to the guidance of cell populations in the developing embryo. By definition, collective cell migration concerns groups of cells that move differently from individual cells of the same type. For the purpose of this review, we have therefore not considered populations of cells directed purely by global signals or periodically arranged local signals (‘Ratchetaxis’ [4]), in which the interactions of cells are negligible.

In our own work, we have mainly used a particular modelling approach in which cells are represented by discrete entities, and the chemical signal is modelled as a continuous field, determined by the solution of a partial differential equation. Such models have the strength that they can easily incorporate individual properties of cells and are computationally straightforward to implement. In the context of the neural crest, with tens to hundreds of cells, such discrete cell-based models also seem more biologically realistic than considering continuous concentrations of cells. The weakness of this type of model framework is the lack of rigorous mathematical theory that would allow us to determine how the various assumptions we make influence the resultant system behaviour (e.g. the boundary conditions or initial conditions imposed). Similarly, we cannot easily obtain analytical expressions on how robust the results are to changes in parameter values. These two drawbacks are less severe in our case, because the model is still computationally cheap to run, enabling us to numerically explore these properties in some depth. Deriving continuum descriptions, in which cells are represented as densities, would allow more analytically rigorous, global parameter analysis and general classification of the model behaviour, but at the cost of difficulty in including cell-level properties. Some progress in this direction has been made [16,17].

While we have focused on theoretical contributions in this review, let us once again emphasize that we advocate the use of models integrated with (not instead of) experiments. It is important to realize that the modelling approach should be chosen appropriate to the question to be answered, and thus different modelling approaches may be applied to answer different questions, just as different experimental systems and techniques are appropriate for different investigations. The range of existing mathematical models of collective migration illustrates how different modelling frameworks are suitable for different experimental systems. In our work [49–51], it was crucial to have off-lattice descriptions (with cell positions not confined to a grid) of cell migration to represent multicellular streaming (as opposed to sheet or chain migration, for example). While neural crest chain migration has been modelled using computationally less extensive grid-based or on-lattice models [9,41,88], streaming migration needs to be represented using off-lattice models to capture realistic cell arrangements and migratory morphologies (as chains and streams can be difficult to distinguish on a lattice). Furthermore, we refrained from studying continuum limits of these cell-level models, as the small number of cells in a leader state strongly motivated a discrete framework with control over individual cell numbers. To give a contrasting example, the migration of the zebrafish lateral line primordium [15] has been modelled both using hybrid [14] as well as continuum [76] approaches, the latter of which is more appropriate than in the neural crest owing to the epithelial nature of the lateral line primordium.

In biology, even the most successful mathematical models are just ‘accurate descriptions of our pathetic thinking’ [21]. Mathematical models (and their computational implementations) are basically logic machines that translate hypotheses into consequences, and do so more consistently and in more complex situations than verbal reasoning (or a conceptual summary ‘model’ found at the end of many papers). As such, models are just an extension of the thought experiment, and while they enable quantitative precision, they do not guarantee it. At the same time, even qualitative insights from models can be useful, as long as we do not confuse *quantitative* with *logically rigorous* (one may even argue that there is no requirement for models to be realistic, as long as they are useful), and bear in mind that models cannot be better than the hypotheses they test.

In summary, there has been an increasing trend in biology to focus on the collection of molecular-level data and detailed intracellular mechanisms. While this strategy has resulted in many successes, we suggest it may not be necessary, or even advisable, to include all known biological detail in a theoretical model. The result can be a system as complex as the real thing, with no real understanding gained. Abstract models and coarse-grained descriptions can help to distinguish the relevant details from the coincidental. Another strength of ‘detail-independent’ models is that they can answer certain questions *despite* a lack of knowledge of biological detail, and thus guide and constrain the subsequent reductionist refinement towards finding molecular mechanisms.

### 4.1. Outstanding biological questions and future theoretical developments

There are several outstanding questions in our understanding of the mechanisms of collective cell migration. We now list some of these questions and possible ways to tackle them as follows.

#### 4.1.1. How do migrating cells interpret guidance signals in the presence of multiple cues?

We have described examples of neural crest cell movements in the presence of a single chemotactic cue, but there probably exist multiple guidance signals within the neural crest microenvironment that cells need to decode to decide in which direction to travel. Fortunately, there are emerging techniques that will allow us to visualize the presence of mRNA and protein of multiple genes within both migrating cells and their microenvironment. These techniques include fluorescence multiplex *in situ* hybridized chain reaction technology [11] and RNAScope [20], which have been developed for use in zebrafish [20], mouse [23] and chick [50]. With these tools, it is now possible to visualize mRNA expression levels of four to five genes in the same tissue, which can be combined with immunohistochemistry to also visualize protein expression levels. One can thus look forward to being able to correlate the *in vivo* spatio-temporal expression patterns of candidate signalling molecules with higher fidelity than current traditional techniques offer. Once the presence or absence of these candidate signalling molecules is determined, we can begin to test the function of these molecules in a combinatorial manner, and integrate with suitably extended mathematical models [59]. This should provide a better understanding of how an individual cell in a population makes a decision to move in a particular direction in the presence of multiple guidance signals.

#### 4.1.2. How is guidance information transferred between cells within the group?

We have described experimental evidence of the local secretion of a chemokine to maintain neural crest cell cohesion [6] and long filopodial protrusions to maintain cell communication [78]. However, we often take for granted that our cells of interest behave as if in a vacuum, without interaction with other cell populations and the underlying substrate. To better understand the transfer of guidance information between cells, we need to clearly visualize the interactions between cells and their local microenvironment (including other cells of the same or different types, and ECM), which may be very dynamic. Newly emerging imaging technologies may now provide a means to better resolve individual cells and fine processes between cells *in vivo*: lattice [10] and two-photon [29,45,82] light sheet microscopy enable three-dimensional imaging of large biological samples much faster and with higher resolution than traditional point scanning light microscopy. When combined with multicolour cell labelling, these tools will be able to distinguish fast cell–cell and cell–substrate dynamics.

#### 4.1.3. Is there a similar set of mechanisms that are altered to produce distinct cell behaviours and patterns?

This is a complex puzzle offered by the wonderful diversity of behaviours and migratory patterns, as displayed for example by neural crest cells, throughout different regions of the embryonic landscape and in different embryo model systems. Alternatively, the different observed migratory patterns between embryo model systems and axial levels may be the result of very distinct biological mechanisms. This offers a particularly challenging question that may be best addressed by the close coordination of multiscale information between model systems including time-lapse and molecular data. As protocols are developed to use advanced imaging tools on many embryo model systems, there is a better chance to cross-analyse time-lapse and genomic datasets, at the level of single cells, to look for patterns in cell trajectories or gene expression that may be common from one species to another during collective cell migration events.

#### 4.1.4. Generalized models to disentangle multiple mechanisms

Advances in our understanding of the mechanisms of collective neural crest cell migration are likely to come from testing the generality of different mechanisms and their interplay at different locations in the body and in different model organisms. Despite the range of evidence for complementary and competing mechanisms for guidance of collective cell migration, we lack an integrated understanding of the interplay of these mechanisms at the population scale. So far, dedicated mathematical and computational models have demonstrated success in case studies focused on particular *in vitro* and *in vivo* experimental settings [6,49–51,53,76,87]. Looking ahead, we think that generalized theoretical descriptions hold the key to map out the spectrum of collective cell migration, by demonstrating how we can combine and reconcile the mechanisms discussed here in a unified framework.

Generalized frameworks may enable us to bridge previous modelling efforts dedicated to particular experimental settings. In the example of the neural crest, cell interactions in previous work range from CiL to contact guidance, and we need to understand how this is determined by cell density [84], confinement [83], microenvironmental properties and the dimensionality of the system. In addition, cell-produced chemokines that facilitate cohesion have been identified in some species [6], whereas in other systems, alternative mechanisms include communication of directional signals through cytoplasmic transfer [48] and trails of breadcrumb-like chemokine deposits [43], while modifications of ECM structure to guide trailing cells may also be important [8]. Distinguishing the effects of these different direct and indirect modes of cell–cell communication on the collective migration using mathematical models ought to be possible, but could run the risk of producing similar collective migration behaviour for large regions of parameter space. Thus, we suspect that mathematical and computational models will need to be more strongly constrained by experimental data if we are to increase their complexity to span the full range of currently debated hypotheses. This will probably come in the form of tracking of all cells in a population in three dimensions, ideally complemented by the live imaging of the distribution of chemical signals.

To conclude, we suggest concrete examples of future theoretical developments: a mathematical model could compare the effects of volume exclusion with contact guidance to those of CiL with CoA, for example, by varying the bias in the direction of a cell's movement. This bias could be towards directions of increasing chemoattractant, as well as towards neighbouring cells, while excluding directions that would lead to overlap of cell bodies. A similar approach has been taken recently by Irons *et al*. [25] to construct agent-based lattice-free models of cell migration with chemotaxis and their corresponding continuum models. Another form of contact guidance is when a cell induces a cell with which it comes into contact to move away, an interaction known as ‘pushing’ [90]. This can be thought of as an asymmetric form of CiL, one which, based on experimental observations, may be more appropriate than symmetric repulsion for the study of multicellular streaming migration, such as in the chick cranial neural crest. Mathematical models could investigate the effect of follow-the-leader contact guidance versus pushing in collective cell migration to shed light on the directionality of communication between cells in moving populations. These are just two of the many ways in which generalized mathematical models could help to increase our theoretical understanding of how constraints and interactions identified shape collective cell migration in development and disease.

## Endnote

1 While our manuscript was under review, a related review article was published by Szabó & Mayor [77], which unfortunately seems to have missed this crucial piece of experimental evidence.
